# Atractylenolide I ameliorated the growth and enzalutamide resistance of castration-resistant prostate cancer by targeting KIF15

**DOI:** 10.1186/s13020-025-01086-1

**Published:** 2025-03-14

**Authors:** Chenglin Han, Bin Yang, Yuxuan Deng, Peng Hu, Bintao Hu, Xiaming Liu, Tao Wang, Chengbao Li, Jihong Liu, Huixing Yuan

**Affiliations:** 1 Department of Urology, Tongji Hospital, Tongji Medical College, Huazhong University of Science and Technology, Wuhan, 430000 China; 2 Department of Anesthesiology, Shandong Provincial Hospital Affiliated to Shandong First Medical University, Jinan, 250021 Shandong China

**Keywords:** CRPC, ATR-I, KIF15, Ubiquitin-proteasomal degradation, AR/AR-V7

## Abstract

**Background:**

Castration-resistant prostate cancer (CRPC) has been a major cause of tumor-associated death among men worldwide. The discovery of novel therapeutic medicines for CRPC remains imperative. Atractylenolide I (ATR-I), a prominent bioactive component from Atractylodes macrocephala, exhibits powerful anticancer potentials in various malignancies. Nevertheless, the ATR-I’s activity on CRPC has not been reported.

**Methods:**

An enzalutamide-resistant (EnzR) cell line was successfully constructed. CCK-8, EdU, wound healing, Transwell assays, flow cytometry, and xenograft tumor models were applied to investigate the antitumor activity of ATR-I against CRPC. The changes in the gene expression profiles after ATR-I treatment were analyzed using RNA sequencing.

**Results:**

ATR-I suppressed the proliferative and migratory abilities of AR^+^ and AR^−^ CRPC cells, while triggering cell cycle arrest and apoptosis. ATR-I also exerted anti-cancer activity on EnzR cell lines. Intriguingly, a combination of ATR-I with enzalutamide synergistically induced more apoptosis of tumor cells. RNA-sequencing identified kinesin family member 15 (KIF15) as a potential target of ATR-I. KIF15 was up-regulated in prostate cancer (PCa), and its higher level was associated with poorer clinical outcomes. Further investigation showed that ATR-I mediated ubiquitin-proteasomal degradation of AR/AR-V7 through targeting KIF15, resulting in CRPC repression. Finally, our in vivo experiment verified that ATR-I alone or in combination with enzalutamide retarded the growth of EnzR xenograft tumors.

**Conclusions:**

These findings identified ATR-I as a promising therapeutic drug for overcoming enzalutamide resistance in CRPC patients and increased our understanding about its antitumor mechanisms.

**Graphical abstract:**

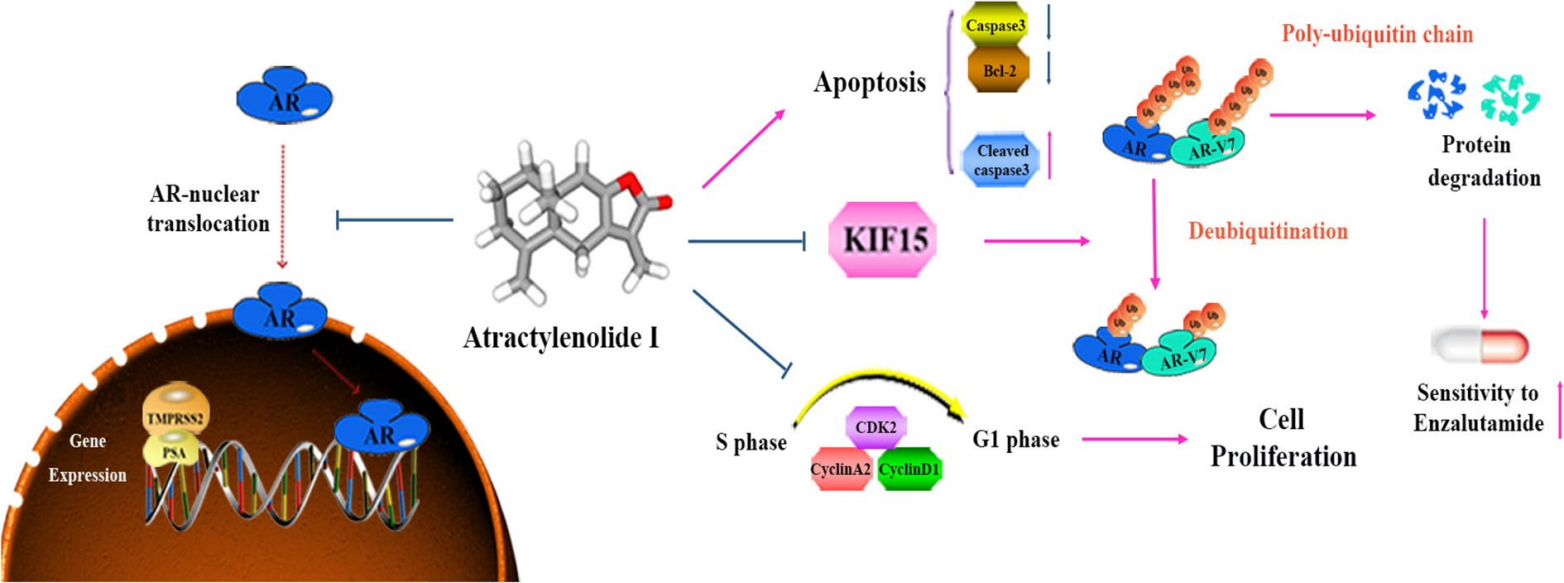

**Supplementary Information:**

The online version contains supplementary material available at 10.1186/s13020-025-01086-1.

## Introduction

Prostate cancer (PCa) is the second-highest morbidity among malignant tumors in men worldwide [1]. Although widespread prostate-specific antigen (PSA) testing has vastly improved the early diagnosis of PCa, ~ 20% of cases represent locally advanced disease, and the 5-year survival rate remains low [2].

Given the importance of the androgen receptor (AR) pathway in PCa development and progression, androgen deprivation therapy (ADT) is perceived as a first-line therapeutic strategy against PCa [3]. Although these advances have provided a temporary respite, most patients inevitably develop a lethal form called metastatic castration-resistant prostate cancer (mCRPC), with a median survival of ~ 3 years [4], which remains a critical clinical challenge. Enzalutamide, an effective AR antagonist, competitively binds to AR and inhibits its nuclear translocation and transcriptional activity [5]. The United States Food and Drug Administration has approved enzalutamide for clinical CRPC treatment, showing impressive overall survival results. Unfortunately, these benefits are limited by primary or acquired drug resistance, which eventually results in therapeutic failure. Therefore, developing therapeutic adjuvants that can be combined with enzalutamide to improve PCa prognosis is crucial [6]. Persistent AR signaling activation is an important mechanism underlying CRPC development and enzalutamide resistance. Numerous studies have identified various genetic alterations at the AR locus, including AR gene amplification and the generation of AR splice variants [7]. Currently, the persistent AR axis is considered a crucial therapeutic target.

Atractylenolide I (ATR-I) is a natural sesquiterpene lactone isolated from *Atractylodes macrocephala* rhizomes. Natural herbal medicines are generally less cytotoxic and cause fewer adverse effects [8]. Over the past two decades, related studies have found that it exhibits multiple biological and pharmacological activities, such as anti-inflammatory [9], antioxidant [10], and antiplatelet activities [11]. Recently, the anti-tumor potential of ATR-I has attracted significant attention. ATR-I reinforces MHC-I-mediated antigen presentation by binding to PSMD4, and enhanced the cytotoxicity of CD8^+^ T cells, thus increasing the responsiveness to immunotherapy [12]. In addition, ATR-I ameliorates tumor cachexia by inhibiting the biogenesis of IL-6 and extracellular vesicles derived from tumor cells [13]. Notably, ATR-I has been shown to reverse tumor-generated immunosuppression by impairing the TLR4/ MyD88/NF-κB pathway [14]. However, little is known about the anti-tumor activity of ATR-I in CRPC cells. In this study, we aimed to evaluate the anti-tumor activity of ATR-I in both AR^+^ and AR^−^ CRPC cells and illustrate the underlying mechanism. Considering the lower cost and slight side effects of Chinese medicine, ATR-I was an alternative and promising prospective anti-tumor agent for CRPC treatment.

## Materials and methods

### Cell culture and human samples

C4-2 and PC-3 cells were purchased from the American Type Culture Collection (ATCC) and cultured in RPMI-1640 or F12K medium supplemented with 10% fetal bovine serum, respectively. To establish C4-2B-EnzR cell lines, C4-2B cells were treated with increasing doses of enzalutamide (MCE, China) from 10 μM to 40 μM for 6 months. Resultant cells were therewith maintained in a medium with 20 μM enzalutamide. All cells were incubated in a humidified 5% CO_2_ environment at 37 °C. In accordance with the Declaration of Helsinki, surgical samples were collected from patients with PCa in the Department of Urology, Tongji Hospital, Tongji Medical College, Huazhong University of Science and Technology (Wuhan, China). Partial samples were frozen in liquid nitrogen for subsequent immunoblotting, and other samples were wrapped in paraffin for preservation.

### Preparation of regents

ATR-I was purchased from the MCE company in the form of drug monomers, and its purity was 99.87%. Enzalutamide was also obtained from MCE.

### Cell viability assay

The appropriate cells were seeded in 96-well plates and treated according to the indicated scheme. 100 μL serum-free culture media with 10 μL CCK8 reagent (Boster, China) was added into each well for 2–4 h. The absorbance values were detected with a microplate reader at 450 nm. Each group was performed with 5 repetitions.

### 5-Ethynyl-2ʹ-deoxyuridine (EdU) assay

After incubation with EdU work solution for 2–4 h, cells were fixed by 4% paraformaldehyde for 15 min and permeabilized with 0.5% Triton X-100 for 10 min. 1 × Apollo^®^ (RiboBio, China) was added to each well for 30 min. At last, cell nuclei were counterstained with Hoechst33342 for 10 min. The EdU-positive cells were calculated under fluorescence microscopy.

### Wound healing assay

After the cell density reaches 80–90%, a straight scratch was formed using the pipette tip. The plate was replaced with a serum-free medium. The photographs were taken at indicated points in time, and the scratch area was calculated using ImageJ software.

### Transwell assay

The lower chamber of Transwell (Corning, America) was filled with 550 μL culture medium containing 10% FBS for PC-3 cells or 20% FBS for C4-2 cells, respectively. A total of 1.5 × 10^5^ C4-2 cells or 4 × 10^4^ PC-3 cells in 200 μL serum-free medium were seeded in the upper chamber. After incubation for 48 h, cells were fixed with 4% paraformaldehyde for 20 min and then stained with 0.1% crystal violet. Five fields were randomly selected to be photographed.

### Flow cytometric analysis

After intervention, cells were collected, washed twice with PBS, and added to 75% ethanol for fixation overnight. Cells were washed with PBS and incubated with RNase (50 μg/mL) and propidium iodide (PI; Yeasen, China) for 30 min. The cycle distribution was detected by flow cytometry.

After intervention, cells were harvested and incubated with 100 μL binding buffer containing 5 μL Annexin V‑FITC (Yeasen, China) and 10 μL PI for 30 min at room temperature in the dark. Flow cytometry was used for detection of cell apoptosis, and all data were analyzed using FlowJo software.

### RNA extraction and quantitative real-time PCR (qRT-PCR)

Total RNA from cells was extracted by SteadyPure Universal RNA Extraction Kit (Accurate Biology, AG21017, China) following the manufacturer’s instructions. The purified RNA (1 μg) from each sample was reverse transcribed into cDNA with Evo M-MLV RT Premix for qPCR (Accurate Biology, AG11706, China). The primers for detection were listed in Table 1. Finally, qRT-PCR was performed using the SYBR Green Premix Pro Taq HS qPCR Kit (Accurate Biology, AG11701, China) to detect mRNA levels. GAPDH was used as an endogenous control, and the relative expression of candidate genes was calculated using the 2^−ΔΔCt^ method [15].

**Table 1:** Primers used in this study

Gene	Forward	Reverse
KIF15	CTGAAGCCTATCAGGTGTTGTC	AGGGAGGTCCGTATATTCACAAT
AR	GCTGCTCCGCTGACCTTA	ATACTGCTTCCTGCTGCTGTT
AR-V7	GAAGCTGCAAGGTCTTCTTCAA	GGTCTGGTCATTTTGAGATGC
TMPRSS2	GTCCCCACTGTCTACGAGGT	CAGACGACGGGGTTGGAAG
PSA	CGCAAGTTCACCCTCAGAAGGT	GACGTGATACCTTGAAGCACACC
GAPDH	GCACCGTCAAGGCTGAGAAC	TGGTGAAGACGCCAGTGGA

### Western blotting

Total proteins from cells or tissues were extracted by RIPA lysis buffer (Beyotime, China) with protease and phosphatase inhibitors. Nuclear and cytoplasmic fractions were isolated using a Nuclear-Cytosol Extraction Kit (APPLYGEN, China). After protein samples containing 1 × loading buffer were boiled and denatured, equal proteins were separated by SDS-PAGE and transferred to PVDF membranes. Whereafter, these bands were interacted with indicated primary antibodies and HRP-conjugated secondary antibodies, followed by visualization using enhanced chemiluminescence solution. The intensity of protein bands was calculated using Image J software.

### RNA sequencing and bioinformatics analysis.

C4-2B-EnzR cells were administrated with ATR-I, and the total RNA was collected by Trizol for transcriptional sequencing (Generead, China). The cut-off value of differentially expressed mRNA was set as log2 |fold change (FC)|> 1 and p-value < 0.05. Based on these differentially expressed genes, enrichment of biological themes was explored using Gene Set Enrichment Analysis (GSEA). Three pairs of samples were provided in this experiment. Single-sample GSEA (ssGSEA) analysis was conducted with the GSVA R package to acquire a hallmark gene set score. Based on TCGA database, the correlation of KIF15 expression with disease-free survival (DFS) and the AR mRNA expression was evaluated from the GEPIA website.

### Immunoprecipitation (IP)

The indicated cells were lysed in NP40 buffer (Boster, China) with protease and phosphatase inhibitors on ice. Lysates were centrifuged at 14,000 rpm for 10 min, and the supernatant was incubated with indicated antibody at 4 °C overnight with rotation. Thereinto, isotype-matched IgG was employed as a negative control. The immune complex was incubated with A/G magnetic beads (MCE, China) for 2 h at room temperature. The mixture was washed with IP buffer three times, supplemented with 1 × loading buffer, and then boiled for 10 min. The immunoprecipitate was separated by SDS-PAGE.

### Cell transfection

When the cell density reached 30–40% or 60–70%, the small interfering-KIF15 (si-KIF15, Genomeditech) or KIF15 plasmid (Tsingke Biotech) was respectively delivered into target cells using lipofectamine™ 3000 reagents (Thermo Fisher Scientific, America) following the manufacturer’s instructions. Prepare two tubes for each well and add corresponding volumes of Opti-MEM in each tube. Then, combine the liquid containing lipo3000 in the first tube and the solution containing si-KIF15 in the last tube, and let stand for 10–15 min. For plasmid transfection, both KIF15 plasmid and p3000 were added into the second tube. After the transfection of 72 h, cells were harvested for efficiency evaluation and subsequent functional assays. Si-KIF15 sequence was displayed in Table 2.

**Table 2:** The sequence of siRNA against KIF15

siRNA	Sequence
Si-KIF15	GGAACAAAUGAGUGCUCUUTT

### Immunohistochemistry

IHC was tested as previously described [16]. The tissue sections were incubated with indicated primary antibodies overnight at 4 °C. In this study, primary antibodies were anti-KIF15 (1:200, Protein Tech, China), anti-Ki67 (1:4000, Protein Tech, China), anti-AR (1:400, Cell Signaling Technology, America), and anti-AR-V7 (1:100, Abcam, Britain). These sections were sequentially treated with biotinylated secondary antibody and horseradish peroxidase (HRP)-conjugated streptavidin. Finally, the slides were stained with chromogenic DAB, followed by counterstaining with hematoxylin. The pictures were taken by light microscopy.

### Mouse xenograft models

Six-week-old male BALB/c nude mice were randomly assigned into four groups (6 mice per group). A total of 4 × 10^6^ C4-2B-EnzR cells in a 200 μL mixture of PBS/Matrigel (1:1, v/v) were injected subcutaneously into the right flank of mice. Tumor growth curves were recorded every 4 days, and tumor volume was calculated as 0.5 × length × width^2^. 28 days later, mice were sacrificed; meanwhile, tumors were weighted and fixed with 4% paraformaldehyde after resection.

### TUNEL assay

TUNEL staining was performed according to the manufacturer’s protocol (Vazyme, America). Tumor tissue specimens were incubated with Proteinase K for 10 min, followed by treatment with Equilibration buffer for 30 min. Then, the slides were incubated with TUNEL reaction buffer for 1 h at 37 °C and the nucleus was dyed blue with DAPI. Cell apoptosis was visualized using the fluorescence microscope.

### Statistical analyses

Statistical analyses were conducted using GraphPad Prism. Data were presented as the mean ± SD in our research. Student’s t-test or one-way ANOVA was applied to analyze the differences between groups. P < 0.05 was considered statistically significant.

## Results

### ATR-I inhibited the proliferation and metastasis of CPRC cells

**Fig. 1:**
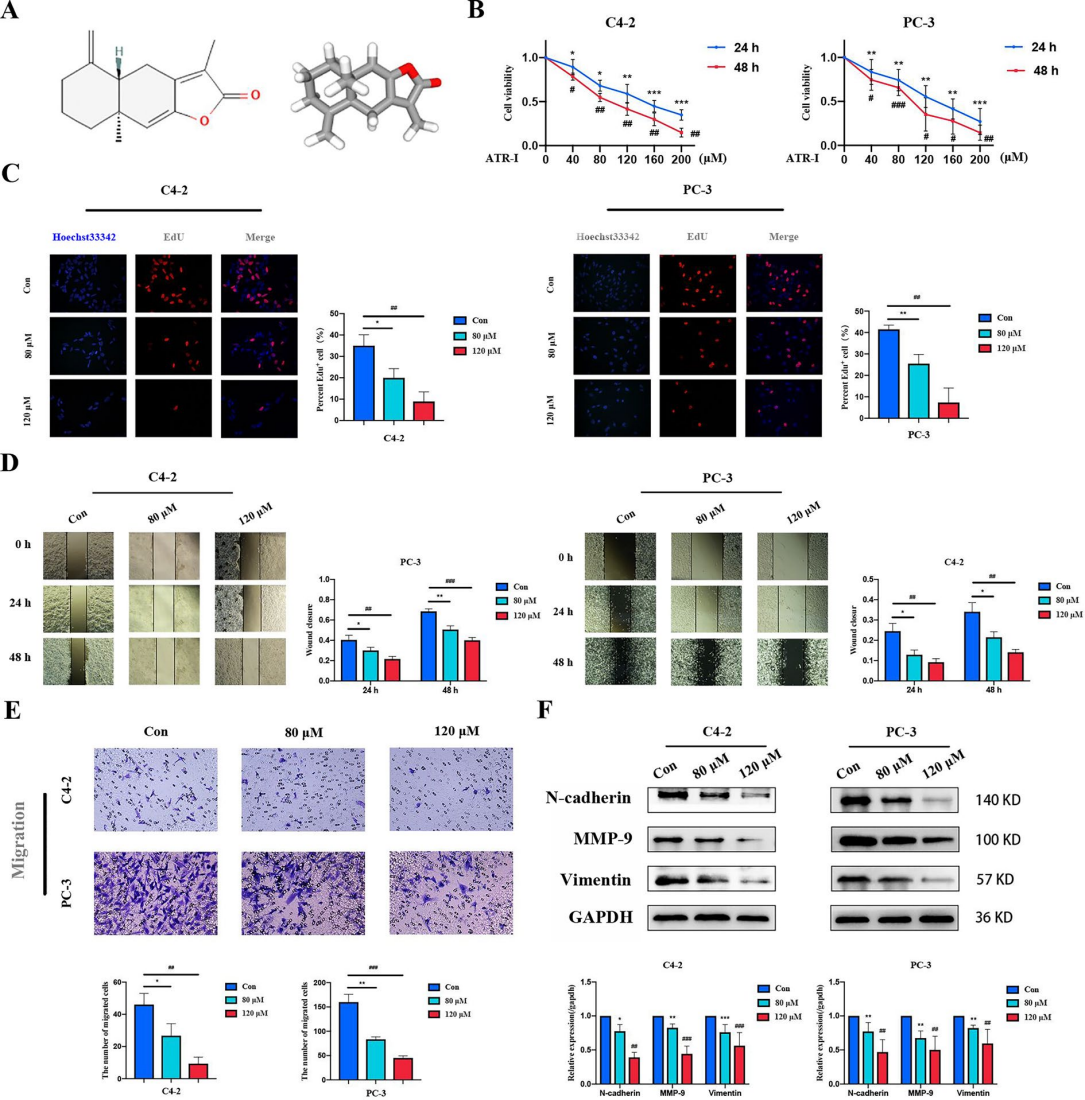
The effects of ATR-I on cell viability, proliferation, and metastasis of CRPC cells. **A** The chemical structure of ATR-I was obtained from the PubChem database (https://pubchem.ncbi.nlm.nih.gov/compound/5321018). **B** Cell viability analysis of C4-2 and PC-3 after treatment with indicated concentrations of ATR-I for 48 or 72 h. **C** EdU staining of proliferated cells. **D** The metastatic ability of CRPC cells was assessed by wound healing assay. **E** Representative images of Transwell results in each group. **F** The expression of genes related with epithelial–mesenchymal transition (EMT) was measured by western blotting (n = 3, *p < 0.05; **p < 0.01; ***p < 0.001; ^#^ p < 0.05; ^##^p < 0.01; ^###^p < 0.001)

The two- and three-dimensional chemical structures of ATR-I are shown in Fig. 1A. To investigate the effects of ATR-I on CRPC, we used two CRPC cell lines (AR-positive C4-2 and AR-negative PC-3). CCK-8 assay results suggested that ATR-I inhibited the viability of PCa cells in a concentration- and time-dependent manner (Fig. 1B). The inhibitive concentration of ATR-I at 50% (IC50) was calculated (Table 3). Based on the above results, we selected 80 μM and 120 μM ATR-I treatment for 48 h in the follow-up experiments. Consistently, ATR-I significantly reduced the percentage of EdU-positive cells in both cell lines (Fig. 1C). In addition, the wound-healing rate was slower in the ATR-I treatment group than in the control group (Fig. 1D). This outcome was also confirmed via a Transwell assay, which suggested that ATR-I dramatically impaired the metastatic capability of CRPC cells (Fig. 1E). Accordingly, western blotting results verified that ATR-I intervention resulted in a lower expression of N-cadherin and Vimentin proteins. Expression of MMP9, a vital migration and invasion marker, also decreased following ATR-I treatment (Fig. 1F).

**Table 3:** IC50 values of ATR-I against tested two cell lines

Cell lines	C4-2		PC-3	
Time (h)	24	48	24	48
IC50 (μM)	139.5	92.83	129.5	91.78
95%CI (μM)	126.1–155.9	83.87–101.9	112.3–149.3	74.21–109.3

*IC50* 50% inhibitive concentration, *CI* confidence interval

### ATR-I induced cell cycle arrest and apoptosis

**Fig. 2:**
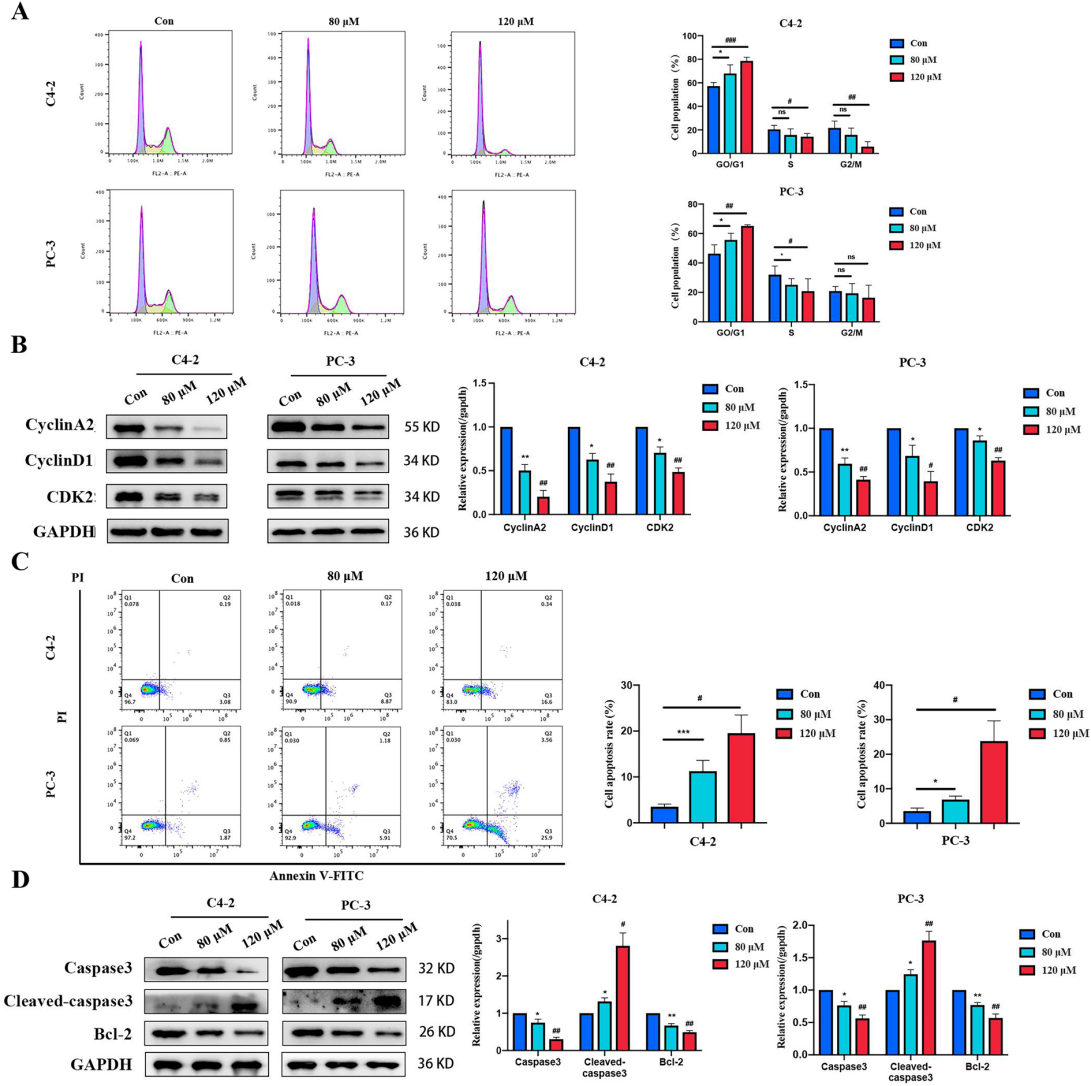
The effects of ATR-I on cell cycle and apoptosis of CRPC cells. **A** The distribution of cell cycle was detected using flow cytometry and the changes in its proportion were presented. **B** The changes in the expression of CyclinA2, CyclinD1 and CDK2 proteins. **C** The apoptosis rate was tested using flow cytometry. **D** The differences in the Caspase 3, Cleaved-caspase 3 and Bcl-2 expression in each group (n = 3, *p < 0.05; **p < 0.01; ***p < 0.001; ^#^ p < 0.05; ^##^p < 0.01; ^###^p < 0.001)

Owing the inhibitory effects of ATR-I on CRPC cell growth, we performed cell cycle assays. Statistical analysis indicated that ATR-I stimulation led to the accumulation of PCa cells in the G0/G1 phase and a corresponding decrease in the population of cells in the S and G2/M phases (Fig. 2A). Western blotting revealed that ATR-I inhibited the expression of several cell cycle-related proteins, such as CyclinA2, CyclinD1, and CDK2 (Fig. 2B). The Flow cytometry results also suggested that the percentage of apoptotic CRPC cells increased remarkably after ATR-I treatment (Fig. 2C). We further examined the effects of ATR-I on apoptosis-related proteins. As illustrated in Fig. 2D, ATR-I reduced the expression of Caspase3 and Bcl-2 while upregulating Cleaved-caspase3 in both types of CRPC cells. However, no significant differences were observed in the expression of Bax. Our results collectively validate that ATR-I inhibits the survival of CRPC cells by arresting the cell cycle in the G1 phase and triggering pro-apoptotic activity.

### ATR-I suppressed the growth of enzalutamide-resistant cells

**Fig. 3:**
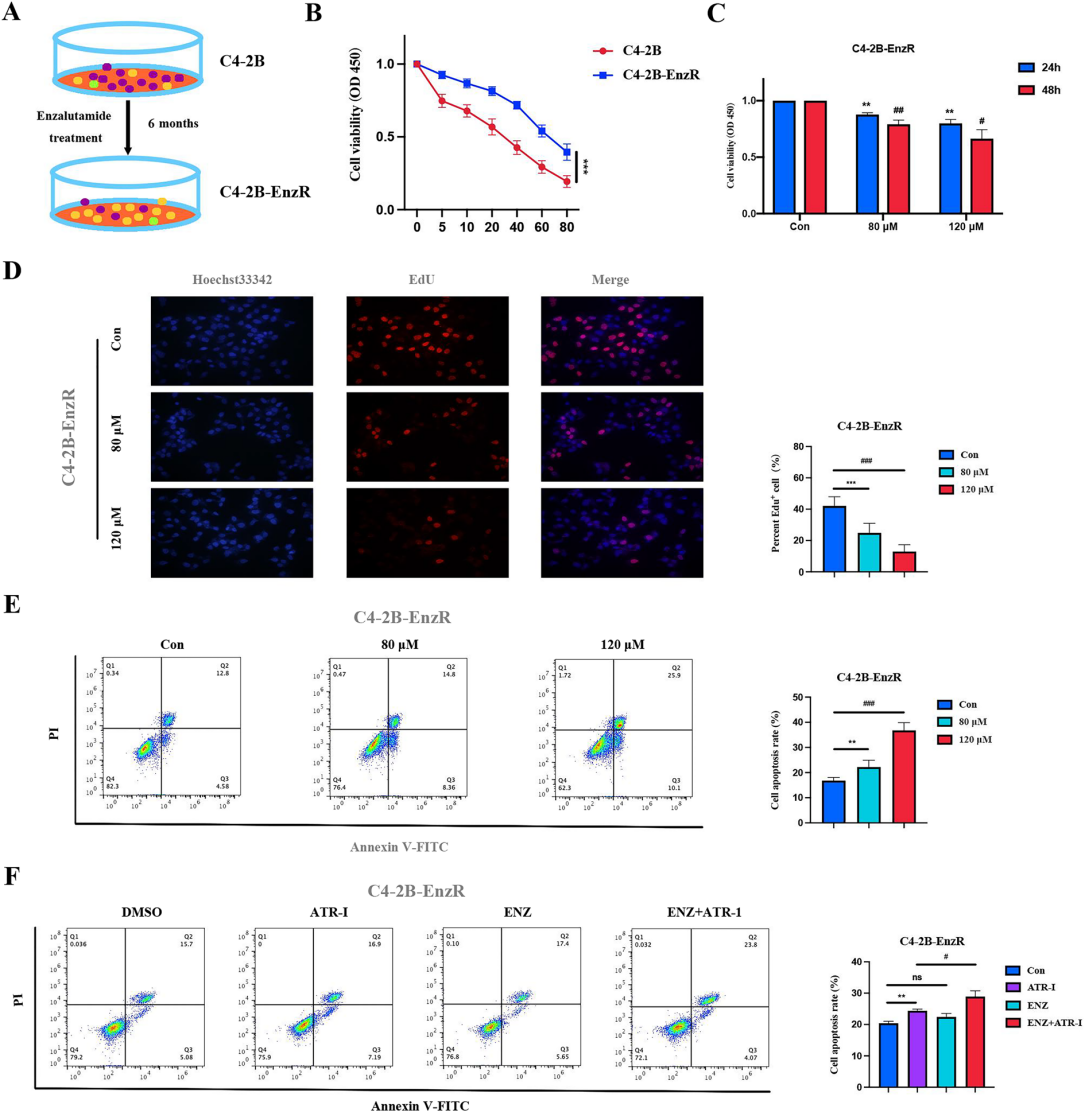
ATR-I inhibited the malignant behaviors of enzalutamide-resistant cells. **A** Construction of enzalutamide-resistant cells. **B** The response of C4-2B and C4-2B-EnzR cells to enzalutamide was detected by CCK8 assay. **C** The effect of ATR-I on the cell viability of C4-2B-EnzR cells. **D** EdU staining for evaluating the antiproliferative potential of ATR-I against C4-2B-EnzR cells. **E** Flow cytometry was conducted to assess the pro-apoptotic activity of ATR-I against C4-2B-EnzR cells. **F** The apoptosis of C4-2B-EnzR cells following ATR-I treatment in combination with enzalutamide (n = 3, **p < 0.01; ^#^ p < 0.05; ^##^p < 0.01; ^###^p < 0.001; ns: no significance). *ENZ* enzalutamide

Enzalutamide reportedly prolongs the survival time of patients with CRPC [17]. Unfortunately, these tumors eventually develop enzalutamide resistance. We first constructed the enzalutamide-resistant C4-2B-EnzR cell line according to a previously reported method (Fig. 3A) [18]. As shown in Fig. 3B, the sensitivity of resistant cells to enzalutamide was notably lower than that of parental cells. The IC50 values of enzalutamide were 23.10 and 66.74 μM for the two tested cells, respectively (Table 4), suggesting that the model was successfully constructed. Subsequently, we conducted the CCK-8 and EdU assays to evaluate the anti-tumor properties of ATR-I against C4-2B-EnzR cells. The results revealed that ATR-I suppressed cell viability and proliferation (Fig. 3C, D). Flow cytometry also showed that ATR-I exerted cytotoxic effects on C4-2B-EnzR cells (Fig. 3E). Intriguingly, enzalutamide alone had no effect on C4-2B-EnzR cells; however, enzalutamide in combination with ATR-I vastly promoted cell apoptosis, indicating that ATR-I and enzalutamide had synergistic therapeutic effects on enzalutamide-resistant cells (Fig. 3F).

**Table 4:** IC50 values of enzalutamide against two tested cell lines

Cell lines	C4-2B	C4-2B-EnzR
IC50 (μM)	23.10	66.74
95%CI (μM)	20.49 – 25.95	60.66 – 74.83

*IC50* 50% inhibitive concentration, *CI* confidence interval

### ATR-I mediated proteasomal degradation of AR/AR-V7

**Fig. 4:**
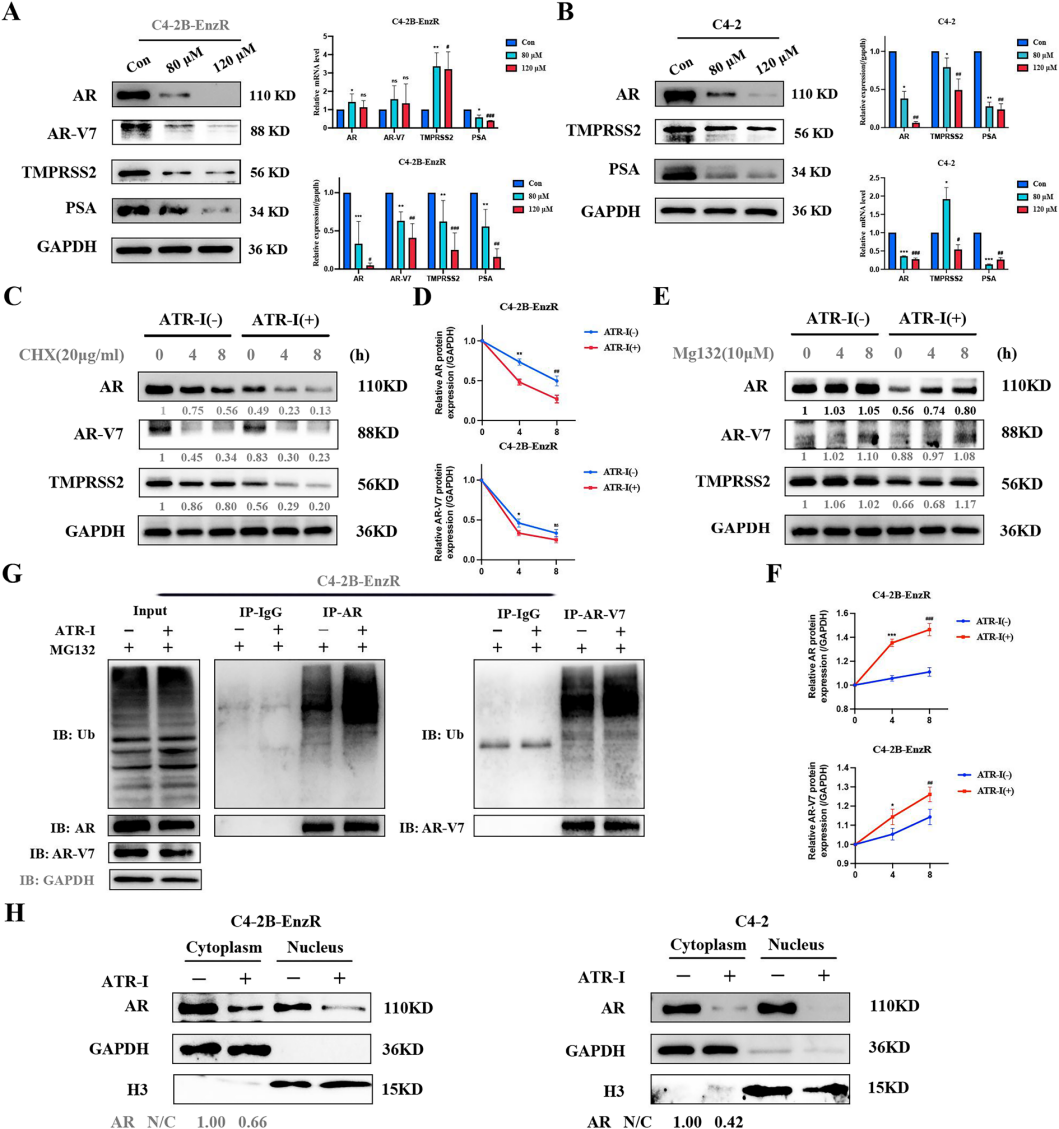
ATR-I inhibited the stability of AR/AR-V7 proteins by promoting their ubiquitination. **A**, **B** Western blotting and qRT-PCR were used to detect the levels of AR, AR-V7, TMPRSS2, and PSA. **C**, **D** The half-lives of AR, AR-V7, and TMPRSS2 were detected in C4-2B-EnzR cells incubated with 20 µg/mL CHX for the indicated times. **E**, **F** The changes in AR, AR-V7, and TMPRSS2 expression in C4-2B-EnzR cells incubated with MG132 (10 μM) for the indicated times. **G** IP assays for detecting the ubiquitination levels of AR and AR-V7 proteins in C4-2B-EnzR cells. **H** The nuclear-cytoplasmic ratio of AR was analyzed using western blotting (n = 3, ***p < 0.001; ^#^ p < 0.05; ^##^p < 0.01; ^###^p < 0.001; ns: no significance). *CHX* cycloheximide

Reactivation of the AR signaling pathway and generation of the AR variant AR-V7 facilitate CRPC occurrence and the development of enzalutamide resistance. In this study, we demonstrated that ATR-I reduced the protein levels of AR/AR-V7 and its downstream target genes, TMPRSS2 and PSA, in C4-2 and C4-2B-EnzR cell lines. In addition, ATR-I treatment resulted in decreased AR mRNA expression in C4-2 cells, whereas the mRNA levels of AR/AR-V7/TMPRSS2 increased slightly in C4-2B-EnzR cells (Fig. 4A, B). To determine whether ATR-I disturbs the stability of AR/AR-V7/TMPRSS2, we treated C4-2 and C4-2B-EnzR cells with cycloheximide (CHX) in the presence or absence of ATR-I. ATR-I led to a sharper decrease in the quantified AR/AR-V7/TMPRSS2 levels compared to the control group, suggesting that their half-lives were shortened after ATR-I treatment (Fig. 4C, D) (Supplementary Fig. 1A, B). The AR/AR-V7 proteins were subjected to ubiquitin–proteasome-mediated degradation [19, 20]. Our results showed that ATR-I-mediated AR/AR-V7 protein downregulation was restored after treatment with the proteasome inhibitor MG132, indicating that ATR-I induced ubiquitin-dependent degradation of AR/AR-V7 proteins (Fig. 4E, F) (Supplementary Fig. 1C, D). In line with the above results, IP assays demonstrated that ATR-I enhanced the degree of AR/AR-V7 ubiquitination in both cell lines (Fig. 4G) (Supplementary Fig. 1E). Additionally, AR and AR-V7 expression was further suppressed following the combination treatment with ATR-I and enzalutamide (Supplementary Fig. 1F). Because AR initiates the transcription of downstream target genes only by moving into the nucleus, we next determined whether ATR-I influences the distribution of AR. Western blotting revealed that the nuclear-to-cytoplasmic ratio of AR dramatically decreased after ATR-I treatment, indicating that ATR-I impaired AR nuclear translocation (Fig. 4H).

### KIF15 is a potential target gene

**Fig. 5:**
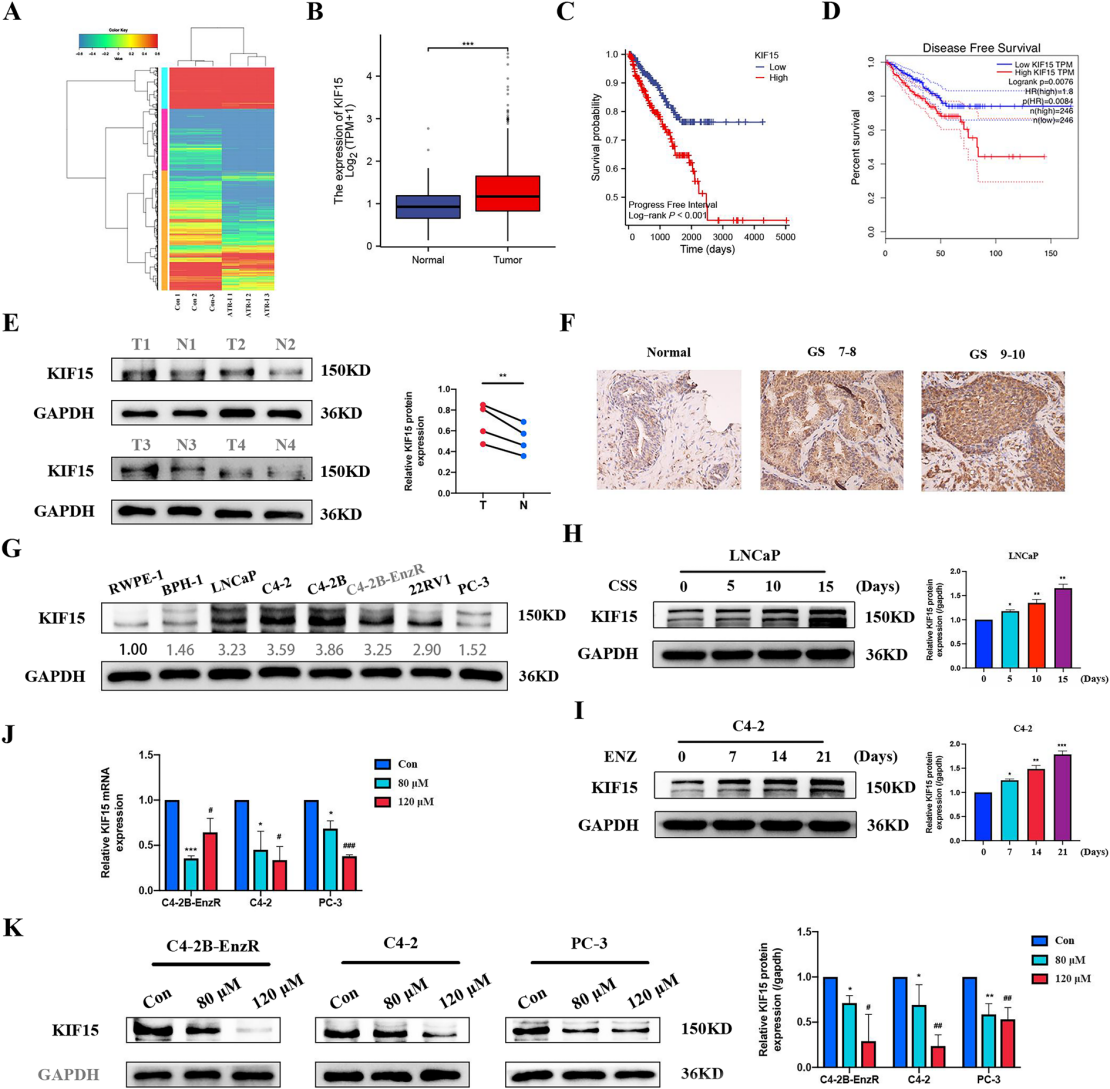
KIF15 was highly expressed in PCa, and ATR-I suppressed KIF15 expression. **A** The heat map revealed the differentially expressed genes between the control and ATR-I treatment groups. **B** The analysis of KIF15 expression in prostate tumor and normal tissues using bioinformatic technology. **C**, **D** Survival curves of PFS and DFS in patients with different KIF15 expression. **E** Differences in the expression of KIF15 protein in four pairs of PCa and its adjacent tissue samples. **F** Immunohistochemistry staining against KIF15 among different GS groups. **G** Differences in the expression of KIF15 protein between the normal prostate cell lines and various PCa cell lines. **H** The KIF15 expression was detected in LNCaP cells cultured in 5% CSS medium for indicated times. **I** The KIF15 expression was tested in C4-2 cells following 20 μM treatment for indicated times. **J**, **K** The changes in KIF15 mRNA and protein expression after ATR-I treatment at different concentrations (n = 3, *p < 0.05; **p < 0.01; ***p < 0.001; ^#^ p < 0.05; ^##^p < 0.01; ^###^p < 0.001). *PFS* progress-free survival, *DFS* disease-free survival, *GS* Gleason score, *CSS* charcoal-stripped serum

RNA sequencing (RNA-seq) was conducted to analyze changes in the gene expression profiles of ATR-I-treated C4-2B-EnzR cells, as revealed in Fig. 5A. GO and KEGG enrichment analyses indicated that the most significant changes were in the expression of genes associated with steroid biosynthesis and metabolism (Supplementary Fig. 2A, B). RNA-seq analysis identified KIF15 as a potential target of ATR-I. Bioinformatics analysis revealed that KIF15 was highly expressed in PCa tissues compared to normal prostate tissues, and KIF15 expression was positively associated with the Gleason score (GS) and pathologic T/N stage (Fig. 5B) (Supplementary Fig. 3A–C). Additionally, higher KIF15 expression predicted poorer progress-free survival (PFS) and disease-free survival (DFS) (Fig. 5C, D). Subsequently, PCa specimens were collected for immunoblotting and immunohistochemical staining. Consistent with the bioinformatics results, KIF15 was overexpressed in PCa tissues, and KIF15 staining intensity gradually increased as GS increased (Fig. 5E, F). Western blotting also demonstrated that KIF15 levels were dramatically higher in most PCa cell lines than in benign cells (Fig. 5G).

KIF15 may play a vital role in CRPC progression and enzalutamide resistance. Using the GSE32269 database, we observed a higher KIF15 level in CRPC than in primary PCa (Supplementary Fig. 3D). Meanwhile, the results of the GSE150807 and GSE151083 datasets indicated that KIF15 was highly expressed in enzalutamide-resistant PCa cells compared to parent cells (Supplementary Fig. 3E, F). As expected, long-term castration deprivation or enzalutamide treatment led to the upregulation of KIF15 expression (Fig. 5H, I). In contrast, silencing KIF15 inhibited the viability of C4-2B-EnzR cells and increased their sensitivity to enzalutamide (Supplementary Fig. 3G). These results show that KIF15 may be a vital driving gene related to PCa progression. However, ATR-I was sufficient to inhibit the mRNA and protein expression of KIF15 in three cell lines (Fig. 5J, K).

### ATR-I inhibited the stabilization of AR/AR-V7 by regulating KIF15 expression.

**Fig. 6:**
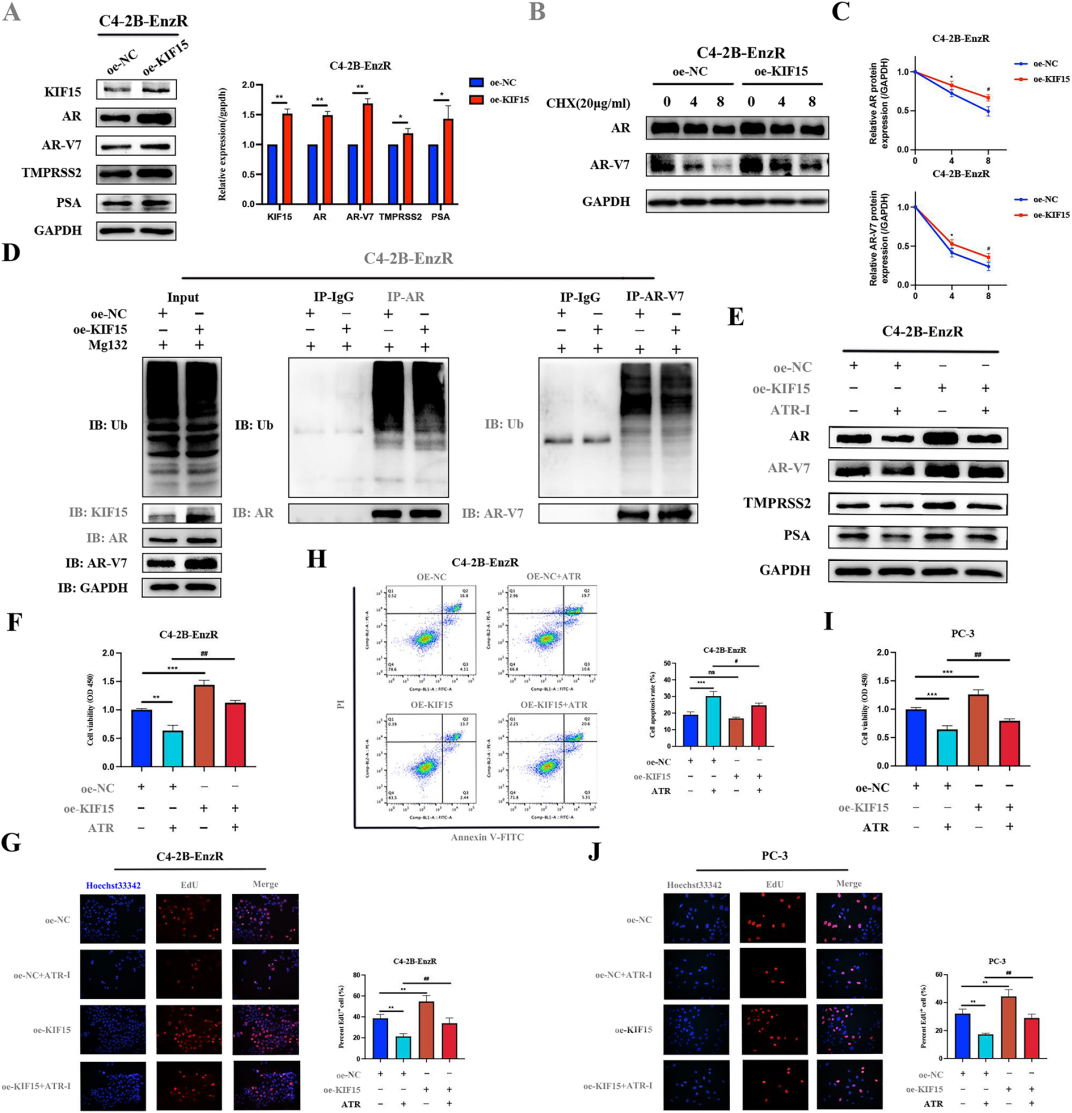
KIF15 overexpression could reverse the inhibitory effects of ATR-I on CRPC. **A** The effects of KIF15 overexpression on the levels of AR, AR-V7, TMPRSS2, and PSA proteins. **B**, **C** The dynamic changes in the AR/AR-V7 expression were evaluated after KIF15 overexpression under the condition of CHX administration. **D** Ubiquitylation of AR and AR-V7 proteins was examined in C4-2B-EnzR cells infected with KIF15 plasmid. **E** The effects of ATR-I combined with KIF15 overexpression on AR and AR-V7 expression. **F**–**H** Differences in cell viability, proliferation, and apoptosis of C4-2B-EnzR cells among different groups. **I**, **J** Comparison of cell viability and proliferation ability of PC-3 cells among different groups (n = 3. *p < 0.05; **p < 0.01; ***p < 0.001; ^#^ p < 0.05; ^##^p < 0.01; ^###^p < 0.001). *CHX* cycloheximide

Single-sample gene set enrichment (ssGSEA) analysis showed the high relevance of KIF15 to tumor proliferation, DNA replication, and the PI3K/Akt/mTOR pathway (Supplementary Fig. 4A–C). Although there was a positive correlation between the mRNA levels of KIF15 and AR from the GEPIA website, no changes in AR/AR-V7 mRNA expression were observed after KIF15 overexpression (Supplementary Fig. 4D, E). Intriguingly, KIF15 upregulation extended the half-lives of AR/AR-V7 proteins, thereby increasing their expression levels (Fig. 6A–C). In line with the above results, our data demonstrated that KIF15 repressed the ubiquitination of AR/AR-V7 proteins (Fig. 6D). Notably, the inhibitory effect of ATR-I on AR/AR-V7 expression was partially rescued by KIF15 overexpression, indicating that ATR-I impaired the stabilization of AR/AR-V7 by downregulating KIF15 (Fig. 6E). We performed a series of biological experiments to further explore whether the anti-tumor properties of ATR-I were dependent on KIF15 downregulation. CCK-8 and EdU assays revealed that ATR-I attenuated the proliferation of C4-2B-EnzR cells, which was partially restored by KIF15 overexpression (Fig. 6F, G). Moreover, the increased KIF15 expression markedly blocked the apoptosis-promoting activity of ATR-I (Fig. 6H). Similarly, KIF15 overexpression partially alleviated ATR-I-induced inhibitive effects on cell survival in AR-negative PC-3 cells (Fig. 6I, J). These data demonstrate that the downregulation of KIF15 is a vital mechanism by which ATR-I exerts its tumor-suppressive functions.

### ATR-I repressed tumor growth in vivo

**Fig. 7:**
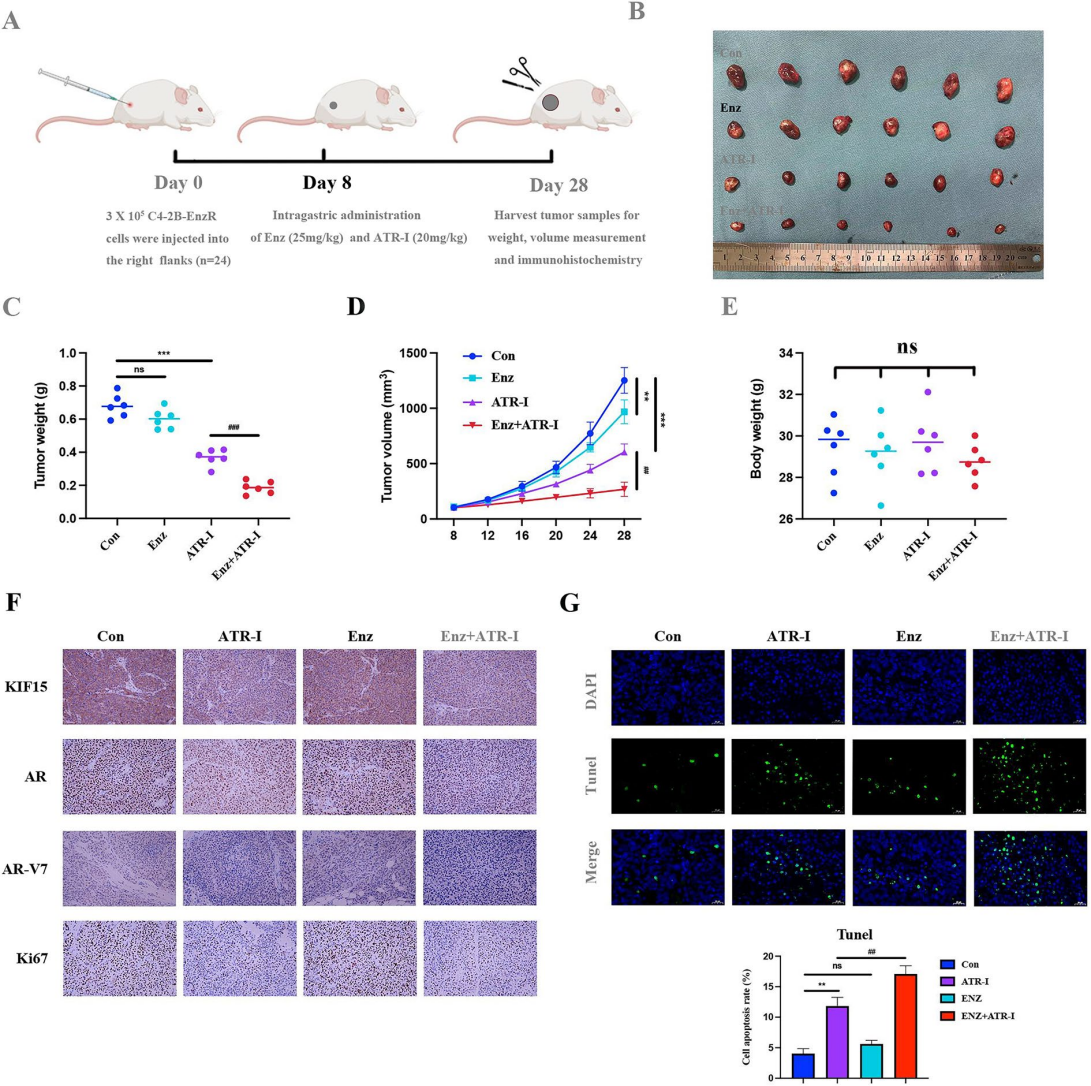
ATR-I inhibited the growth of CRPC in vivo. **A** The dosage regimen against xenografted tumors. **B** Tumors were isolated and pictured. **C** The weight of exfoliated tumors was detected. **D** Tumor volume was measured every 4 days. **E** Body weight-time curve in mice. **F** Immunohistochemistry staining of KIF15, Ki67, AR, and AR-V7 in each group. **G** The apoptosis rate of tumor cells in tissues was analyzed by TUNEL assay (n = 6, **p < 0.01; ***p < 0.001; ^##^p < 0.01; ^###^p < 0.001; ns: no significance). *ENZ* enzalutamide

To determine the influence of ATR-I on PCa development in vivo, we constructed xenograft models via the subcutaneous injection of C4-2B-EnzR cells (Fig. 7A). ATR-I treatment alone inhibited tumor growth, and the combination of ATR-I and enzalutamide had an obvious synergistic effect (Fig. 7B). The volume and weight of subcutaneous tumors remarkably decreased after ATR-I administration for ~ 1 month (Fig. 7C, D). Conversely, there were no significant differences in mice body weight between groups, suggesting that short-term ATR-I treatment had no obvious toxic side effects (Fig. 7E). As shown in Fig. 7F, Ki67, a typical proliferative marker, was dramatically reduced after ATR-I treatment and was further inhibited when combined with enzalutamide. Additionally, the changes in AR/AR-V7 expression were consistent with the Ki67 variation trend observed in the treatment groups. We also noted that combination treatment with ATR-I and enzalutamide significantly exacerbated tumor cell apoptosis (Fig. 7G). Taken together, these results indicate that ATR-I is a potential adjuvant for enzalutamide.

## Discussion

PCa is one of the most common malignancies in males all over the world [21]. Although most early-stage patients with PCa have satisfactory survival after ADT, 20% of these patients eventually progress to the CRPC stage, with a higher mortality [22]. Chemotherapeutic drugs inevitably produce cytotoxic adverse effects. In recent years, there has been a gradual upward trend in the morbidity and mortality of PCa despite significant improvements in comprehensive treatment, which brings a huge challenge to the healthcare system. These situations urgently required novel pharmacotherapies to prevent the progression of CRPC.

Natural products with diverse bioactivities have been an invaluable resource in drug discovery. ATR-I has presented anti-cancer potential in various adenocarcinomas, such as gastric cancer [23], colon cancer [24], and breast cancer [25]. However, little is known about the detailed effects of ATR-I on CRPC.

KIF15, a member of the kinesin-12 subfamily, is a tetrameric spindle motor protein that drives microtubule-dependent plus-end motion [26]. During mitotic spindle assembly, KIF15 acts as a Ki67-interacted partner to expedite spindle elongation and centrosome separation [27]. Therefore, dysregulation of KIF15 may result in aberrant cell division, differentiation, and tumorigenesis. Accumulating evidence suggests that KIF15 is often upregulated in multiple malignancies and plays a vital role in tumor progression. For instance, aberrant KIF15 expression promotes pancreatic cancer growth by activating the MEK/ERK signaling pathway [28]. However, KIF15 knockdown inhibites the development of endometrial cancer by restraining the Wnt/β-catenin signaling [29]. A previous study reported that KIF15 expression was increased in CRPC and KIF15, at least in part, facilitates the proliferation of CRPC cells in an EGFR-dependent manner [30]. Overall, we rationalize KIF15 as a novel driver of CRPC progression. Our study demonstrated that KIF15 was highly expressed in PCa, and higher KIF15 expression hinted at shorter OS and DFS. Additionally, KIF15 expression was correlated with GS and pathological T/N stages. RNA-seq analyses identified KIF15 as a potential target of ATR-I, which was in agreement with the qRT-PCR and western blotting results. Subsequent rescue experiments demonstrated that KIF15 overexpression reduced the anti-tumor efficacy of ATR-I against AR^+^ and AR^−^ CRPC cells, indicating that ATR-I suppressed the CRPC progression through targeting KIF15.

Enzalutamide, a next-generation AR inhibitor, has acquired a certain therapeutic effect against CRPC; however, this disease ultimately develops resistance to enzalutamide, leading to a mean survival time of 16–18 months [31]. Phenotypic experiments have shown that ATR-I alone exhibits anti-tumor activity against enzalutamide-resistant cells and synergizes with enzalutamide to restrict the survival of enzalutamide-resistant cells in vitro and in vivo. Most mechanisms underlying enzalutamide resistance depend on the continuous activation of AR signaling. Notably, the AR-full length is well expressed in most enzalutamide-resistant CRPC that has a lower expression of canonical androgen response element-dependent AR target genes, such as KLK3, KLK2, and TMPRSS2. Depletion of AR-full length suppresses the survival of these cells, indicating that AR remains functionally critical for the acquisition of ENZ resistance [32, 33]. A subset of noncanonical AR-binding sites (ARBS) with gained AR binding intensity is critical for the growth of CRPC cells by activating the expression of ID1, PFN2 and ID3 [34]. Current views support that the expression and transcriptional activity of AR are augmented in CRPC despite enzalutamide treatment [35]. AR reactivation is triggered by AR gene amplification, AR point mutations, and tumor-intracrine androgen synthesis [36]. Moreover, the generation of AR-spice variants (AR-Vs) responsible for enzalutamide resistance has attracted considerable attention. After undergoing a process of alternative splicing, AR is converted to a truncated isoform that lacks the ligand-binding domain in the carboxy-terminus. However, AR-Vs remain constitutively active and enable tumor cells to survive even in the absence of androgens. AR-V7, the most common variant, significantly increased during ADT and enzalutamide therapy [37]. A preclinical study demonstrated that detectable levels of AR-V7 in circulating tumor cells (CTCs) at baseline contribute to the maintenance of PSA levels in enzalutamide-treated samples [38]. Thus, AR-V7 levels can be used to evaluate the response of patients with CRPC to enzalutamide treatment. The combination of ATR-I and enzalutamide synergistically suppressed AR and AR-V7 expression, providing a plausible explanation for ATR-I being a sensitizer of enzalutamide.

The balance between protein synthesis and degradation contributes to the maintenance of cellular homeostasis. In this study, ATR-I was effective in suppressing the expression of AR and AR-V7 proteins in both two cell lines but did not affect their mRNA levels in C4-2B-EnzR cells. This difference may be attributed to genetic changes. In the culture of C4-2B-EnzR derived from C4-2B cells, certain ATR-targeted transcription factors responsible for regulating AR and AR-V7 transcription may be missing. Additionally, the binding activity of transcription factors varies at different stages of disease development. For example, genome-wide AR Chip-seq profiling revealed that 12,652 ARBS were lost in C4-2-EnzR compared to C4-2, whereas 6908 ARBS were obtained. CXXC5 and TET2 were overexpressed in C4-2-EnzR cells compared to parent cells, which caused increased AR-binding intensity at ARBS-gained CpGi loci, thereby forming a non-canonical AR addiction [34]. On the other hand, we inferred that KIF15 might regulate AR and AR-V7 expression at the post-translational level. As expected, ATR-I accelerated proteasome-dependent degradation of AR/AR-V7 proteins in both cell lines. The degradation of AR and AR-V7 proteins is heavily dependent on the ubiquitin–proteasome system (UPS). As a post-translational regulatory mechanism of proteins, ubiquitination is a multi-cascade reaction process. Ubiquitin, a highly conserved 76-amino acid polypeptide, is activated by the ubiquitin-activating enzymes (E1s) in an ATP-dependent manner. Subsequently, activated ubiquitin is transferred to ubiquitin-conjugating enzymes (E2s) to form a connection via thioester bonds. Third, ubiquitin ligases (E3s) catalyze the transfer of ubiquitin from E2s to specific substrates. The proteasomal substrate is conjugated to a poly-ubiquitin chain using a specific E3 ligase, which is then recognized by the 26S proteasome for degradation [39]. Ubiquitination is a highly reversible process in which deubiquitinating enzymes (DUBs) release conjugated ubiquitin from substrates to reverse ubiquitin signals. E3 ligases (Skp2, SIAH2, and MDM2) and DUBs (USP14 and USP22) are associated with AR and AR-V7 protein degradation [40, 41]. Compared to traditional AR antagonists, targeted AR degradation seems to be more effective in inhibiting AR signal activation [42]. Various novel drugs have been designed to specifically induce AR and AR-V7 degradation using the proteolysis-targeting chimera (PROTAC) technology. PROTAC is a heterobifunctional molecule consisting of a target protein ligand and an E3 ubiquitin ligase ligand that promotes the binding of E3 ligase to the target protein and catalyzes the ubiquitination-dependent degradation of substrates [43]. ARV-110, developed by Arvina company using PROTAC technology, was the first AR degradant agent in clinical trials. It induces 95–98% AR protein degradation by promoting the interaction of the E3 ligase CRBN with AR, significantly restraining the growth of PCa [44].

Recently, it has been reported that KIF15 regulates the ubiquitination of several proteins, thus affecting their stability. KIF15 is responsible for USP10-induced PGK1 deubiquitination, thereby enhancing the glycolytic capacity for pancreatic tumor growth [45]. Additionally, KIF15 recruits and promoted the binding between USP15 and DEK, facilitating the proliferation of leiomyosarcoma cells by preventing DEK degradation [46]. Our study showed that KIF15 overexpression decreases the stability of AR/AR-V7 proteins and increases their ubiquitin levels. Additionally, the ATR-I-mediated downregulation of AR and AR-V7 was restored by KIF15 overexpression. An earlier study reported that KIF15 positively regulates the stability of AR/AR-V7 proteins by increasing their bond with USP14 [18]. We speculate that ATR-I promotes AR/AR-V7 degradation by partially suppressing KIF15 expression. The key enzymes involved in this process warrant further exploration. Additionally, the anti-tumor activity of ATR-I was diminished after KIF15 overexpression in AR^+^ and AR^−^ CRPC cells, indicating that KIF15 expedited CRPC progression in both an AR-dependent- and independent manners, such as the EGFR signaling pathway.

This study has some limitation. First, we only explored the alteration of AR-V7; whether ATR-I affected the expression of other AR splicing variants containing intact NTD (such as AR-V567s) remains unclear. Additionally, we revealed that the effects of KIF15 on the stability of AR/AR-V7 proteins were associated with ubiquitination modification; however, the enzymes involved in this process deserve further exploration. Third, the mechanism underlying the contribution of KIF15 to AR^−^ CRPC has yet to be clarified. Nevertheless, this study represents a breakthrough in this field. For the first time, we demonstrated that ATR-I exerts anti-tumor activity in CRPC and revealed the underlying mechanism. In contrast, this study identifies ATR-I as an enzalutamide sensitizer and provides a basis for applying ATR-I in the clinical treatment of CRPC.

## Conclusions

In summary, our results illustrate that ATR-I is an effective anti-tumor herbal medicine against refractory CRPC. ATR-I suppresses the survival and metastasis of CRPC cells and has a synergistic anti-tumor effect with enzalutamide. ATR-I can induce proteasomal degradation of AR/AR-V7 to overcome enzalutamide resistance, which depends in part on its specific inhibitory effect on KIF15 expression. These findings raise the possibility of potential use of ATR-I for the treatment of CRPC, which has great clinical value.

## Supplementary Information

Supplementary Fig. 1. ATR-I promoted the ubiquitin-dependent degradation of AR in C4-2 cells. (A-B) The half-lives of AR and TMPRSS2 were detected in C4-2 cells incubated with 20ug/ml CHX for the indicated times. (C-D) The changes in AR and TMPRSS2 expression in C4-2 cells incubated with MG132 (10 μM) for the indicated times. (E) IP assays for detecting the AR protein ubiquitination. (F) The effects of ATR-I combined with enzalutamide on the expression of AR and AR-V7 proteins (n = 3, **p < 0.01; ***p < 0.001; ^##^p < 0.01; ^###^p < 0.001; ns: no significance) CHX: cycloheximide; ENZ: enzalutamide.

Supplementary Fig. 2. The effects of ATR-I on cellular functions and signaling pathways. (A-B) GO and KEGG enrichment analysis of differentially expressed genes after ATR-I treatment. GO: Gene Ontology; KEGG: Kyoto Encyclopedia of Genes and Genomes.

Supplementary Fig. 3. KIF15 expression was associated with poor prognosis. (A-C) Bioinformatic analysis of the correlations between KIF15 expression and GS, T stage as well as N stage. (D) The differences in KIF15 expression between primary PCa and CRPC based on the GSE32269 database. (E–F) Comparison of KIF15 expression between enzalutamide-resistant PCa cells and their parent cells based on GSE150807 and GSE151083 databases. (G) The response of C4-2B-EnzR to enzalutamide was detected after KIF15 silencing. (n = 5, **p < 0.01; ***p < 0.001; ^###^p < 0.001).

Supplementary Fig. 4. The pathway and tumor hallmarks that were regulated by KIF15. (A-C) KIF15 was involved in tumor proliferation, DNA replication, and PI3K/Akt/mTOR pathway. (C) The positive correlation between the mRNA levels of AR and KIF15 from the GEPIA website. (D) qRT-PCR was used to assess the effect of KIF15 overexpression on AR and AR-V7 expression at the mRNA level. (n = 3, ***p < 0.001; ns: no significance).

## Data Availability

The data are available from the corresponding author upon reasonable request.

## Abbreviations

ATR-I: Atractylenolide I
AR: Androgen receptor
PSA: Prostate-specific antigen
ADT: Androgen deprivation therapy
CRPC: Castration-resistant prostate cancer
IP: Immunoprecipitation
KIF15: Kinesin family member 15
IC50: 50% Inhibitive concentration
CHX: Cycloheximide
GO: Gene Ontology
KEGG: Kyoto Encyclopedia of Genes and Genomes
GS: Gleason score
PFS: Progress-free survival
DFS: Disease-free survival
ssGSEA: Single-sample gene set enrichment
CSS: Charcoal-stripped serum
AR-Vs: AR-spice variants
ARBS: AR-binding sites
CTCs: Circulating tumor cells
UPS: Ubiquitin–proteasome system
DUBs: Deubiquitinating enzymes
PROTAC: Proteolysis-targeting chimera
