# Supplementary figures and images for: Atractylenolide I ameliorated the growth and enzalutamide resistance of castration-resistant prostate cancer by targeting KIF15
Source: Chin Med. 2025 Mar 14;20:35. doi: 10.1186/s13020-025-01086-1 (PMC11909966; doi:10.1186/s13020-025-01086-1)

**A**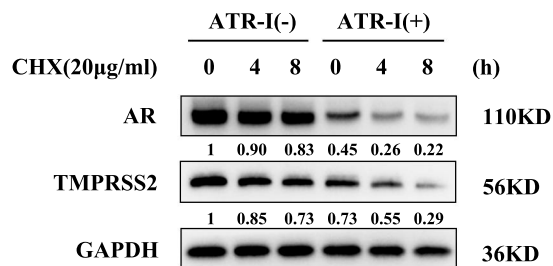**B**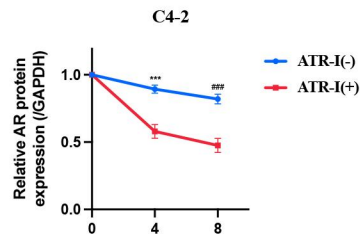**C**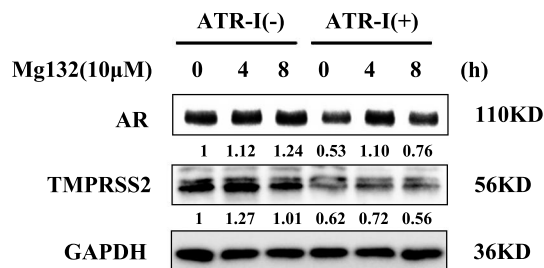**D**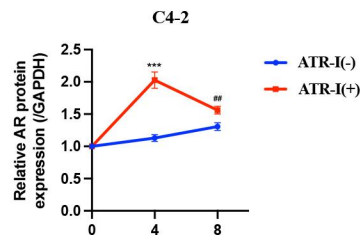**E**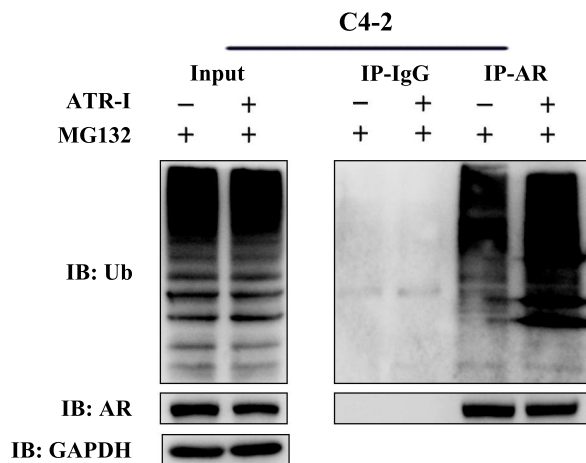**F**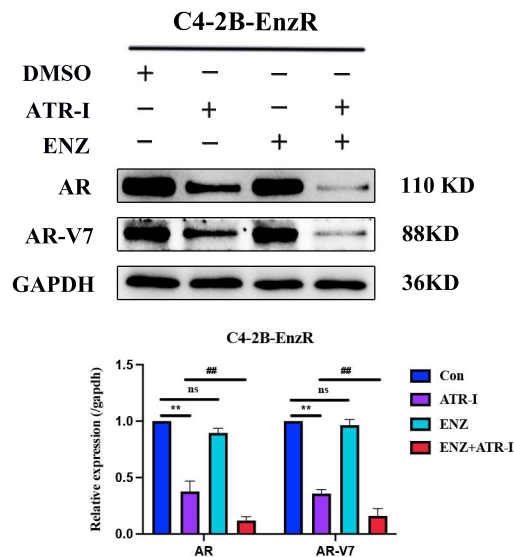

Supplement: Supplementary file 1 — Supplementary Fig. 1. ATR-I promoted the ubiquitin-dependent degradation of AR in C4-2 cells. (A-B) The half-lives of AR and TMPRSS2 were detected in C4-2 cells incubated with 20ug/ml CHX for the indicated times. (C-D) The changes in AR and TMPRSS2 expression in C4-2 cells incubated with MG132 (10 μM) for the indicated times. (E) IP assays for detecting the AR protein ubiquitination. (F) The effects of ATR-I combined with enzalutamide on the expression of AR and AR-V7 proteins (n = 3, **p < 0.01; ***p < 0.001; ##p < 0.01; ###p < 0.001; ns: no significance) CHX: cycloheximide; ENZ: enzalutamide. [file 13020_2025_1086_MOESM1_ESM.pdf]

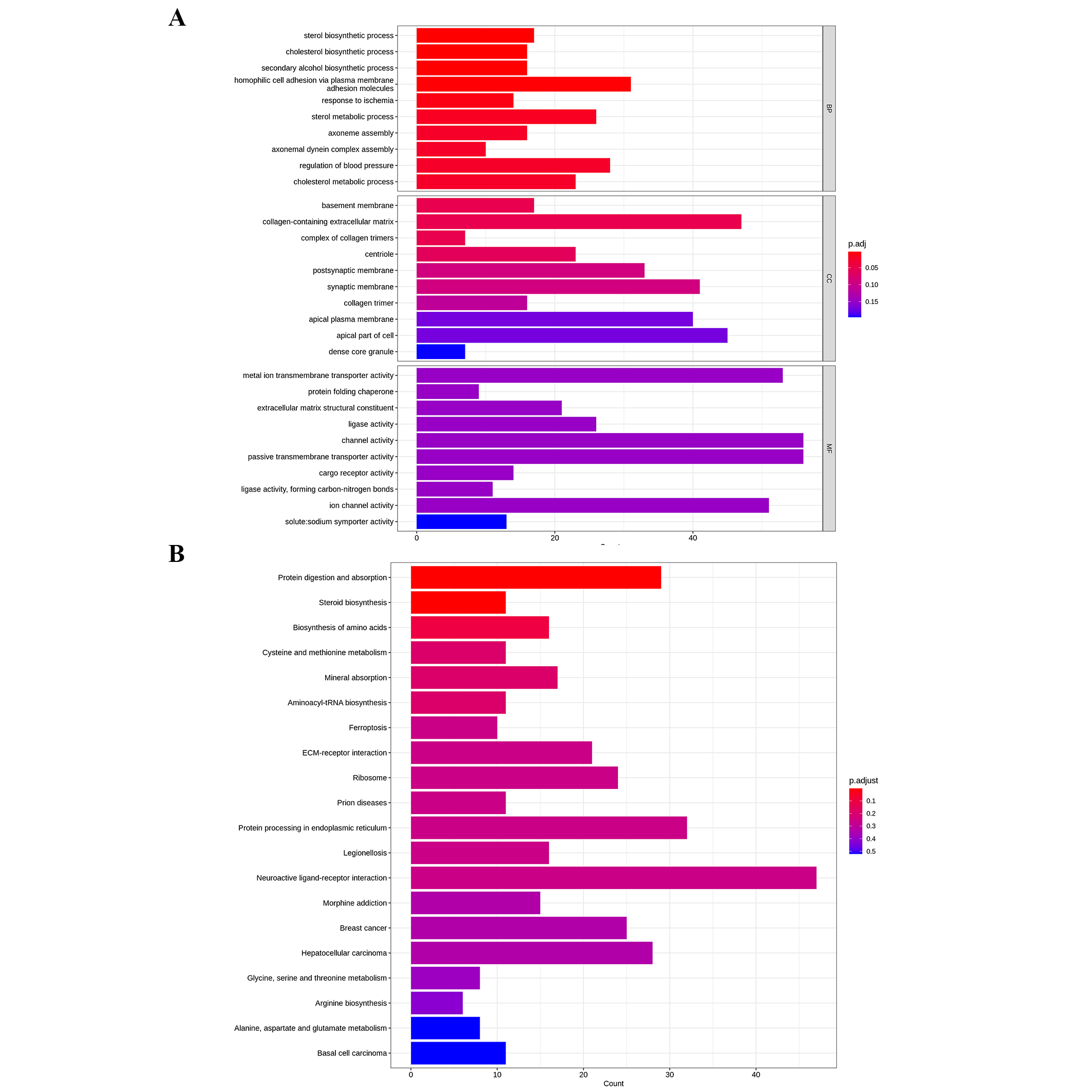

Supplement: Supplementary file 2 — Supplementary Fig. 2. The effects of ATR-I on cellular functions and signaling pathways. (A-B) GO and KEGG enrichment analysis of differentially expressed genes after ATR-I treatment. GO: Gene Ontology; KEGG: Kyoto Encyclopedia of Genes and Genomes. [file 13020_2025_1086_MOESM2_ESM.tif]

**A**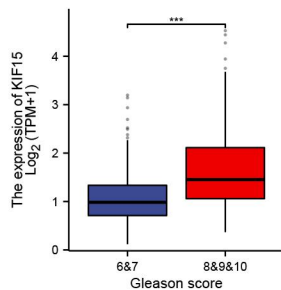**B**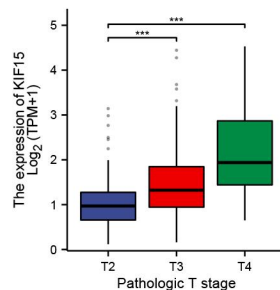**C**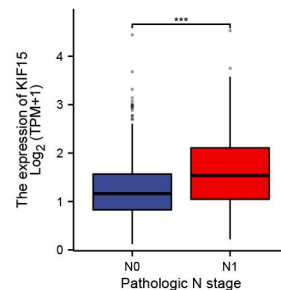**D**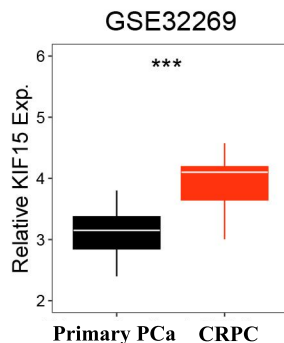**E**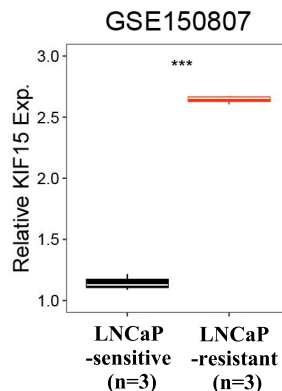**F**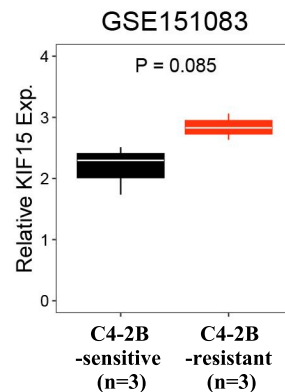**G**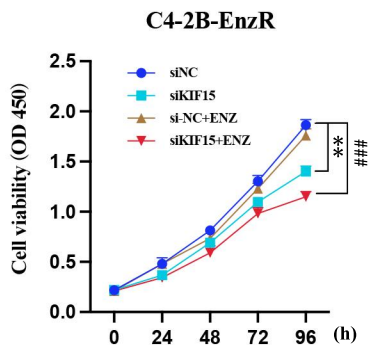

Supplement: Supplementary file 3 — Supplementary Fig. 3. KIF15 expression was associated with poor prognosis. (A-C) Bioinformatic analysis of the correlations between KIF15 expression and GS, T stage as well as N stage. (D) The differences in KIF15 expression between primary PCa and CRPC based on the GSE32269 database. (E–F) Comparison of KIF15 expression between enzalutamide-resistant PCa cells and their parent cells based on GSE150807 and GSE151083 databases. (G) The response of C4-2B-EnzR to enzalutamide was detected after KIF15 silencing. (n = 5, **p < 0.01; ***p < 0.001; ###p < 0.001). [file 13020_2025_1086_MOESM3_ESM.pdf]
