# Supplementary material for: Atractylenolide I ameliorated the growth and enzalutamide resistance of castration-resistant prostate cancer by targeting KIF15
Source: Chin Med. 2025 Mar 14;20:35. doi: 10.1186/s13020-025-01086-1 (PMC11909966; doi:10.1186/s13020-025-01086-1)
Supplement: Supplementary file 4 — Supplementary Fig. 4. The pathway and tumor hallmarks that were regulated by KIF15. (A-C) KIF15 was involved in tumor proliferation, DNA replication, and PI3K/Akt/mTOR pathway. (C) The positive correlation between the mRNA levels of AR and KIF15 from the GEPIA website. (D) qRT-PCR was used to assess the effect of KIF15 overexpression on AR and AR-V7 expression at the mRNA level. (n = 3, ***p < 0.001; ns: no significance). [file 13020_2025_1086_MOESM4_ESM.pdf]

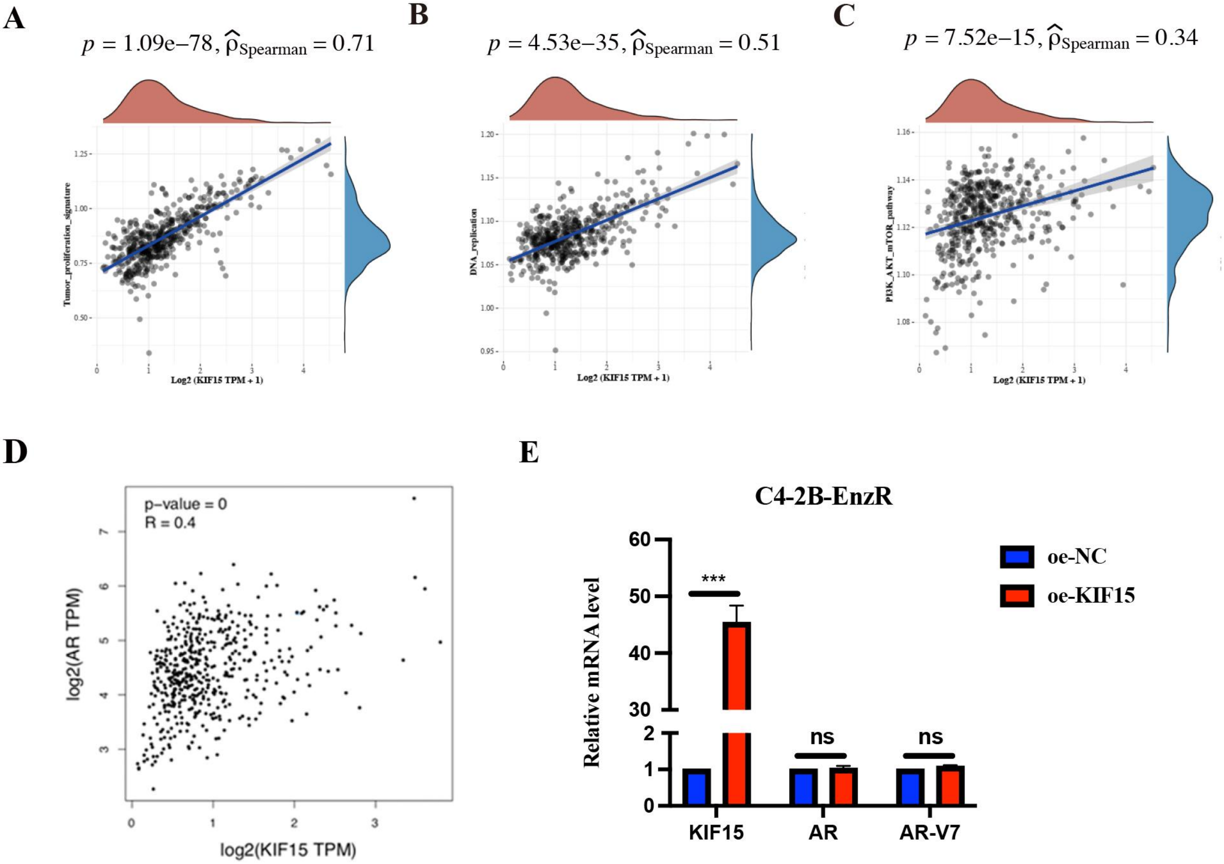

A
p = 1.09e−78, ρ̂ Spearman = 0.71
Tumor_proliferation_signature
Log2 (KIF15 TPM + 1)
B
p = 4.53e−35, ρ̂ Spearman = 0.51
DNA_replication
Log2 (KIF15 TPM + 1)
C
p = 7.52e−15, ρ̂ Spearman = 0.34
PI3K_AKT_mTOR_pathway
Log2 (KIF15 TPM + 1)
D
p−value = 0
R = 0.4
log2(AR TPM)
log2(KIF15 TPM)
E
C4-2B-EnzR
Relative mRNA level
oe-NC
oe-KIF15
***
ns
ns
KIF15
AR
AR-V7
